# Congenital Left Ventricular Aneurysm

**DOI:** 10.1016/j.jaccas.2024.102453

**Published:** 2024-08-21

**Authors:** Eric Bailey, Adam Small, Dan G. Halpern

**Affiliations:** a Department of Medicine, NYU Grossman School of Medicine, New York, New York, USA; b Adult Congenital Heart Disease, Division of Cardiology, Department of Medicine, NYU Grossman School of Medicine, New York, New York, USA

**Keywords:** cardiomyopathy, congenital heart disease, left ventricular aneurysm

## Abstract

This paper presents the case of a 30-year-old man who was diagnosed with an apical-lateral wall left ventricular aneurysm with scarring, prominent left ventricular trabeculations, and mildly diminished systolic function. Working diagnosis was a congenital left ventricular aneurysm in the setting of left ventricular noncompaction, yet with a questionable defect of the pericardium.

## Graphical Abstract

**Figure:**
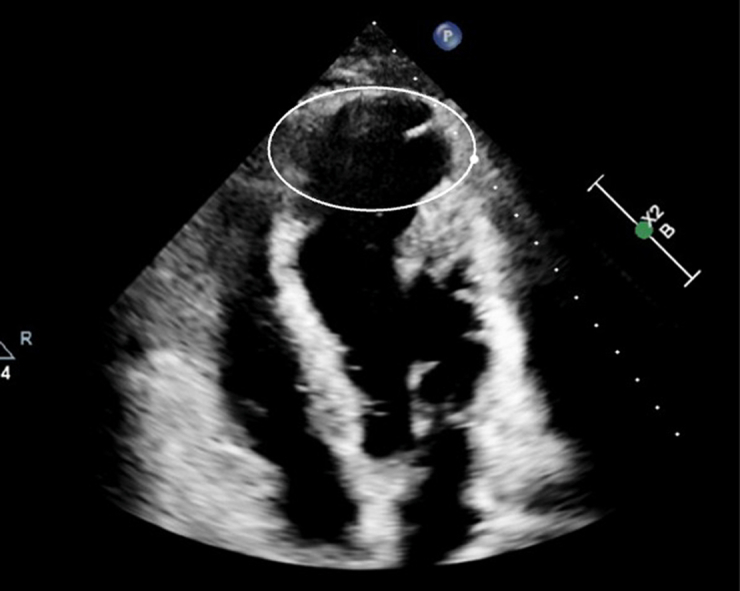

## History of Presentation

A 30-year-old man presenting with atypical chest pain and occasional short-lived palpitations without syncope showed an electrocardiographic abnormality consisting of sinus tachycardia, left ventricular (LV) hypertrophy, and repolarization changes. The patient reported chronic exercise intolerance.

**Learning Objectives**

• To understand that congenital LVAs are a rare abnormality, typically of the apex of the left ventricle; they are a nidus for ventricular arrhythmias.
• To consider neurohormonal blockade initiation given high mortality rates from heart failure in this population.
• To understand that arrhythmia monitoring should be part of the initial evaluation of patients with congenital LVAs.
• To establish that currently the pathophysiology is unclear, *LDB3* and *ZASP* are genes of interest.

**Figure 1:**
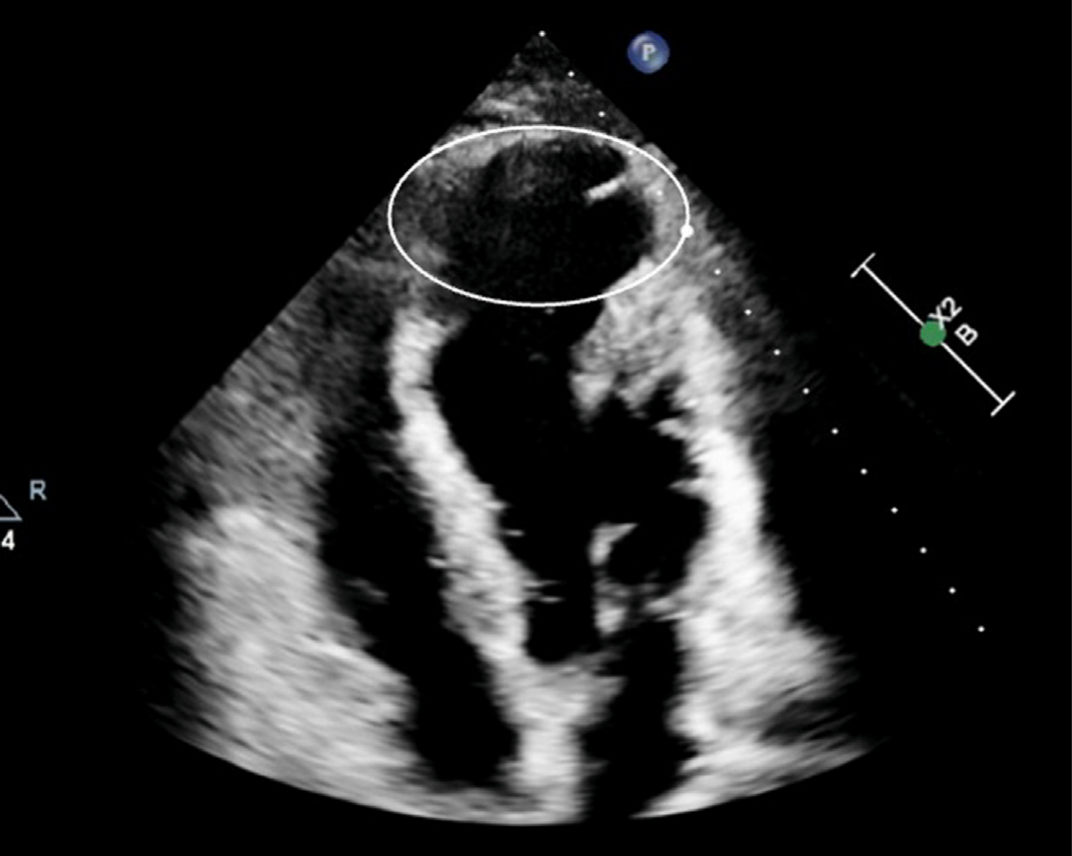
Transthoracic Echocardiogram Axial 4-Chamber View Circle shows prominent papillary muscles and septal protrusions in the left ventricle.

Physical examination revealed normal rate and regular rhythm, normal S1 and S2, and no murmurs, S3, S4, or rubs. Subsequent workup included a transthoracic echocardiogram and revealed an estimated global LV ejection fraction of 45% to 50%, a dyskinetic and aneurysmal apex, and an apical lateral wall (Figure 1).

**Figure 2:**
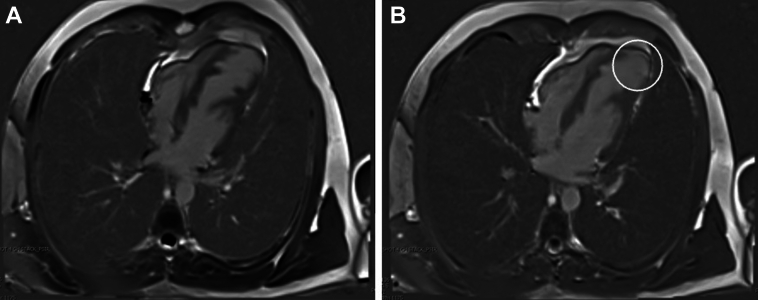
3 Different Slices From the Same Cardiac Magnetic Resonance Sequence (A) Good visualization of the left ventricular septal protrusions and apical myocardial thinning is shown. (B) Circled area shows myocardial thinning and pericardial thinning.

Cardiac magnetic resonance further characterized the aneurysm as a 3.2- × 1.7-cm focal outpouching with wall thinning and severe hypokinesis with a thin layer of subendocardial late gadolinium enhancement of the apical lateral and true apical segments. Prominent trabeculations were seen within the viable part of the LV myocardium (Figure 2).

## Past Medical History

Born at term without known maternal pregnancy complications, the patient reported normal growth during childhood, but had chronically reduced functional capacity and inability to participate in regular exercise activities limited by dyspnea. Additional medical history included anxiety and depression, for which the patient was treated with a selective-serotonin reuptake inhibitor.

## Differential Diagnosis

The differential diagnosis included congenital coronary artery abnormalities, congenital partial absence of the pericardium with ventricular herniation, and congenital cardiomyopathy. The myocardium morphology suggests a congenital myopathy within the LV noncompaction spectrum with course trabeculations, yet the discrete apical-lateral LV aneurysm located in a noncoronary distribution and subendocardial scarring raises the diagnosis of a congenital left ventricular aneurysm (LVA). The dilemma was whether the focal thinning of the pericardium around the aneurysm could also represent a pericardial defect with potential herniation.

## Investigations

**Figure 3:**
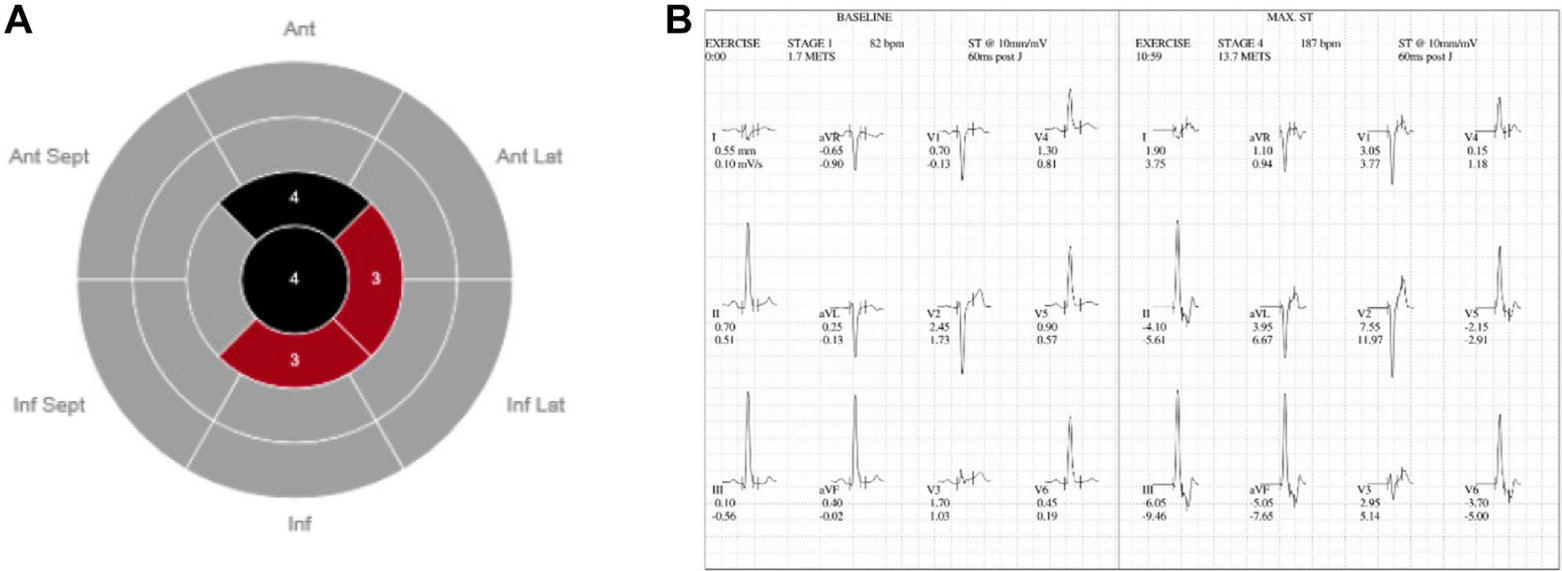
Stress Test Results (A) An absence of uptake was noticed in the apex and apical anterior section of the left ventricle. Additionally, there were severe reductions in uptake in the apical inferior and apical lateral segments. (B) Exercise brought on ST-segment depressions in leads II, III, aVF, V_5_, and V_6_, which all had a normal ST-segment at rest. Ant = anterior; Inf = inferior; Lat = lateral; Sept = septal.

**Figure 4:**
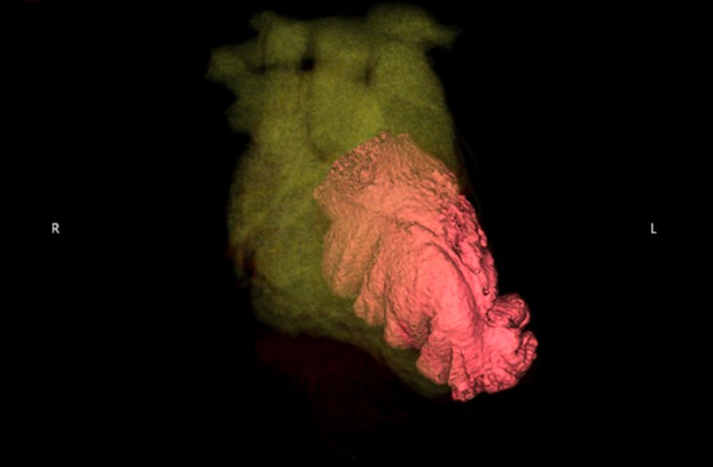
3-Dimensional Images Created From Computed Tomography Angiography The left ventricular external structure is highlighted in red.

Active ischemia was ruled out using a single-photon emission computed tomography exercise stress testing, yet revealed a large size fixed defect at the aneurysmal site seen in Figure 3. Seven-day event monitor showed scattered premature ventricular complexes and an electrophysiology-induced rapid ventricular arrhythmia at the aneurysm site for which the patient received a subcutaneous internal cardioverter-defibrillator (ICD). A computed tomography angiography of the coronaries redemonstrated the unusual myocardial architecture and revealed normal coronary origin and course, without stenosis, yet paucity of coronary distribution to the aneurysm. The pericardium appeared significantly thinned to absent at the aneurysmal site (Figure 4).

**Figure 5:**
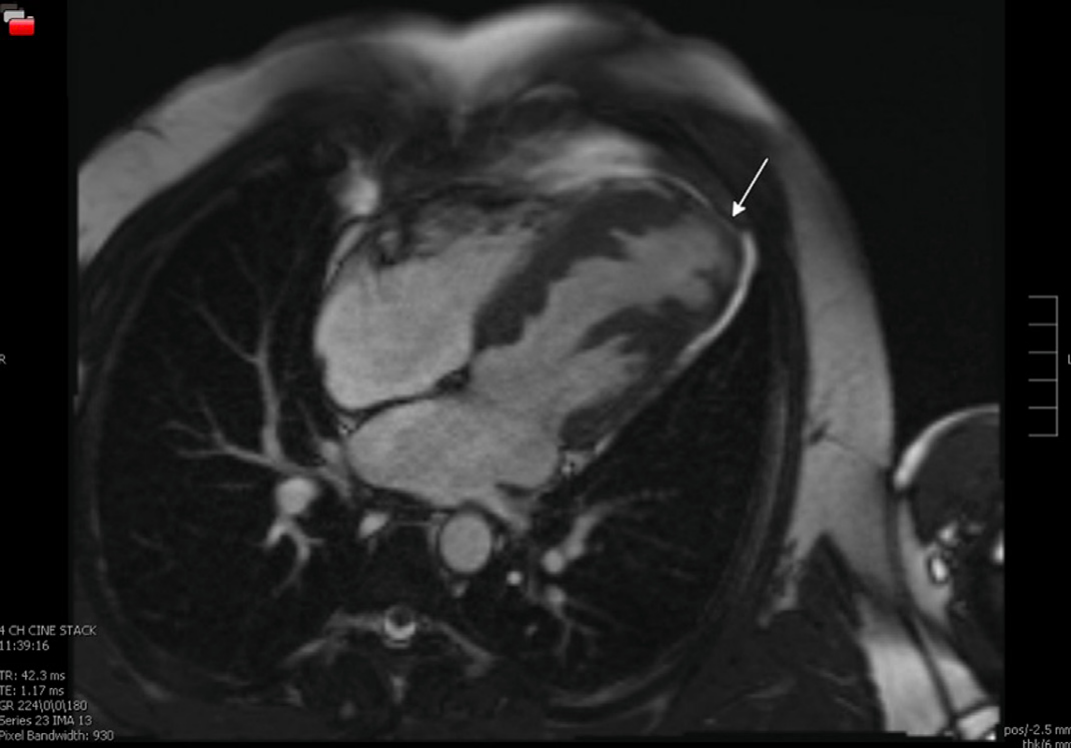
Cardiac Magnetic Resonance With Pericardial Protocol Arrow points toward the area in question, with possible partial pericardial defect.

In addition to LVA, the diagnosis of congenital partial absence of the pericardium was considered, and in consultation with cardiothoracic surgery, the patient underwent video-assisted thoracoscopic surgery for direct pericardial visualization with plan for repair if a defect was found, the area of pericardial and myocardial thinning can be seen in Figure 5. Video-assisted thoracoscopic surgery revealed a thin yet intact pericardium with bulging of the pericardium where the LV apical aneurysm was located. Final considerations included a LVA in the setting of a myopathy within the scope of LV noncompaction, given the prominent trabeculations noted on cardiac magnetic resonance.

## Management (Medical/Interventions)

The patient was initiated on aspirin and neurohormonal blockade including metoprolol succinate, valsartan-sacubitril, and empagliflozin, and had the aforementioned placement of an ICD.

## Discussion

LV structural abnormalities have been reported to develop from the fourth week of embryonic life.^1^ Isolated LVAs are commonly an incidental cardiac finding with a reported history of normal pregnancy, yet LVAs may have catastrophic in utero outcomes presenting with fetal arrhythmia, hydrops fetalis, and fetal demise.^1^ Ohlow et al^2^ reported that among those with LVA, 62% are found in the apex, 15% are in the apical-lateral wall, 8% are lateral, 8% are septal, and 4% are posterolateral. Associated abnormalities are common and include intracardiac shunts, coronary anomalies, and extracardiac thoracoabdominal abnormalities. Histology reveals fibrous replacement of the normal 3-layered myocardium. There are also reports of multilobulated bulging arising from the left ventricle. In a retrospective adult cohort, the prevalence of LVA was found to be 0.34%, commonly diagnosed incidentally, with a male predominance with an occasional arrhythmic presentation.

Ohlow et al^3^ identified several complications in a population of 354 patients with congenital LVA. Ventricular arrhythmias occurred in 18.1%, rupture occurred in 4%, syncope occurred in 8.3%, and embolic events occurred in 4.9% (whereas thrombotic material was present within the aneurysms in 11%).^3^ Despite these potentially catastrophic complications, the diagnosis was typically made during routine workup for other issues and the patients were asymptomatic. The presented patient had noted atypical chest pain, which was reported in this cohort as a composite of both typical and atypical chest pain at 10.2%.^3^ A total of 205 patients had available follow-up data; 26 of which died with the most common causes being congestive heart failure and sudden cardiac death at 50%, sudden cardiac death at 27%, and rupture at 23%.^3^ This emphasizes the importance of neurohormonal blockade and electrophysiologic evaluation in this population with lower threshold consideration of a protective ICD. Speaking to the arrhythmogenicity of these aneurysms, in this case the patient had inducible ventricular arrhythmia within the aneurysm, prompting ICD placement.

Prominent trabeculation has been reported to be associated with LVA. Sato et al^4^ and Shan et al^5^ both reported on cases of congenital LVA with prominent LV trabeculation. Sato et al^4^ proposed that disruptions in the microcirculation during development may facilitate this abnormal development, whereas Shan et al^5^ reported on a mutation in the *LDB3* or *ZASP* gene as a cause of congenital LVA, a gene also implicated in cases of idiopathic dilated cardiomyopathy.^5^^,^^6^ Given reports of prominent LV trabeculation in patients with congenital LVA, the poorly differentiated myocardium in those patients may be serving as a nidus for subsequent myocardial thinning and aneurysm formation during development.

## Conclusions

We presented a diagnostically challenging case of LVA, which is a rare congenital cardiac abnormality that commonly presents incidentally but may be associated with life-threatening complications. High rates of arrythmias and sudden cardiac death in this population should prompt thorough electrophysiological evaluation with consideration of an ICD. Partial absence of the pericardium with LV herniation through the defect may mimic an LVA and should be considered in the differential diagnosis.

## Funding Support and Author Disclosures

The authors have reported that they have no relationships relevant to the contents of this paper to disclose.
